# *PLOS Medicine* at 10 Years: Two Imperatives

**DOI:** 10.1371/journal.pmed.1001749

**Published:** 2014-10-28

**Authors:** The PLOS Medicine Editors

## Abstract

On the occasion of the journal's tenth anniversary, the *PLOS Medicine* Editors reflect on the challenge of attaining universal availability for high-quality medical research.

*Please see later in the article for the Editors' Summary*

Ten years ago this month, in *PLOS Medicine's* first issue the founders of PLOS laid out their vision for the journal:

The established medical publishers have turned their back on the opportunity to make the latest and best medical information available to anyone with an Internet connection. With the launch of *PLOS Medicine*, we are embracing this opportunity. *Everything we publish is immediately, freely available online throughout the world, with no restrictions on distribution, copying, printing, or legitimate use* [1].

In accordance with the founders' statement that “all of today's leading medical journals are more than 70 years old, and *PLOS Medicine* is here to challenge the status quo” [1], the editorial team issued its first challenge in the same issue:

Medical journals have allowed their interests to become aligned with those of the pharmaceutical industry by printing advertisements for drugs, publishing trials designed by drug companies' marketing departments, and making profits on reprints used as marketing tools. *PLOS Medicine* will not accept advertisements for pharmaceutical products or medical devices [2].

The journal has since endeavored to challenge the status quo on an ongoing basis, shining a light on a variety of unhealthy industry practices [3]–[6], breaking the news that most published research is questionable on statistical grounds alone [7], and committing to improve the situation through consistent statistical review of research submissions, promotion of guidelines to improve the quality of research reporting [8],[9], the PLOS-wide policy on data availability [10], and, most recently, journal guidelines to enhance transparency in observational studies [11].

Over the past decade, however, open access to medical research has become more complicated than just choosing an idealistic new journal over regressive old ones. Researchers now face a complex array of hybrid subscription/publication fee models, institutional archiving policies (sometimes entailing university claims of copyright that publishers may or may not accept), funder mandates, data availability requirements, blacklists and whitelists aiming to identify publishing scams appearing in the guise of open-access (OA) journals, and copyright agreements that retain publisher rights under the guise of OA licenses. Scientific publishing is in a feverish state of transition regarding what will be shared and how.

Medical science, perhaps more than any other field caught up in this transition, combines an ethical imperative to ensure the reliability of published research with an ethical imperative to ensure universal accessibility and reuse. In accordance with the first imperative, the medical community values the quality assurance that they may associate with established journals; no responsible professional would wish to base clinical practice or health policy on data of questionable provenance that has been inadequately vetted. On the other hand, traditional indicators of an article's overall importance—in particular, which journal a paper “got into” along a perceived hierarchy of publications—are still confused with, and for some purposes valued higher than, validity or practical relevance. Such indicators are overdue for reassessment, and fulfillment of the second imperative depends on their evolution.

While seeking to publish in the most highly esteemed general medical journals is a long-standing tradition, the misuse of journal-level metrics, particularly the journal impact factor, has led to a number of problems. *PLOS Medicine* was one of the first journals to bring attention to the situation [12], and more than 11,000 colleagues have since called for reform by endorsing the American Society for Cell Biology (ASCB)'s San Francisco Declaration on Research Assessment (DORA) [13]. These problems include the practice of using editorial decisions as proxies for the overall significance of individual research articles and, by extension, for the value of individual authors to their institutions. Furthermore, each of the three general medical journals that boast the highest impact factors has been a prominent landmark in the publishing landscape for well over 100 years. The situation is a setup for stasis: if high impact factors drive high-quality submissions, these few journals, which currently impose and profit from copyright barriers, will have little financial incentive to change their business models to permit full access and reuse. On the contrary, they may continue to keep much of the world's most important medical research, and the data that underlie it, behind access barriers.

If left to traditional publishers, this tangled web of competing interests, copyright barriers, inappropriate metrics, desire for reward and recognition, and misaligned business models could continue to defy the second imperative—that medical knowledge be universally accessible—for years to come. It's worth remembering, though, that ultimately journals can only publish the research that authors submit and only on copyright terms that authors are willing to grant. In a time of declining research budgets and increasing influence on medicine by corporations that see health through the distorting lens of outsize profit taking, publisher-imposed restrictions on access to knowledge may seem just one among several pressing issues that medical researchers face. This issue, though, is one that researchers themselves fully hold the power to solve, if they choose to do so.

As *PLOS Medicine* celebrates its tenth anniversary, the editors renew our aspiration to play a part in making that choice unmistakably clear. We see research ethics and scientific validity as essential to the ultimate goal of better serving patients, and we aim to explore new ways to expedite the availability of clinically important results. We decline to engage in the misuse of metrics that, through entrenched ulterior interests in the publishing industry, work against the ethical imperative to share data and analyses, and we will work instead to establish more appropriate standards in the service of open research.

High-quality medical knowledge is a quintessential public good, and *PLOS Medicine* will continue working with researchers to advance its unrestricted availability. To those authors who took a chance on submitting their work—which they could easily have seen published in established titles—to our first issue, and to those who entrust their finest work to *PLOS Medicine* for freely readable and fully reusable publication today, we extend our sincere and ongoing appreciation.
